# The Lipid-Metabolism-Associated Anti-Obesity Properties of Rapeseed Diacylglycerol Oil

**DOI:** 10.3390/nu16132003

**Published:** 2024-06-24

**Authors:** Yilin Mao, Danhong Zheng, Lin He, Jing Chen

**Affiliations:** 1 JNU-UPM International Joint Laboratory on Plant Oil Processing and Safety, Department of Food Science and Engineering, Jinan University, Guangzhou 510632, China; yilinmaofood@163.com; 2 College of Pharmacy, Jinan University, Guangzhou 510632, China; zdh935882622@163.com; 3 Institute for Advanced and Applied Chemical Synthesis, Jinan University, Guangzhou 510632, China; 4 Fastco Biotech (Hangzhou) Co., Ltd., Hangzhou 311222, China; helin@fastco.cn

**Keywords:** diacylglycerol, rapeseed oil, white adipose tissue, lipid metabolism, gut flora

## Abstract

To investigate the effects of rapeseed diacylglycerol oil (RDG) intake on lipid accumulation and metabolism in C57BL/6J mice, obese mice were fed a high-fat diet in which 45% of the total energy content came from RDG (RDGM group) or rapeseed triacylglycerol oil (RTGM group). This diet intervention was conducted for 12 weeks following the establishment of the obese mouse model. By the end of the experiment, the serum glucose levels of the mice in the RTGM and RDGM groups were 13.0 ± 1.3 mmol/L and 9.7 ± 1.5 mmol/L, respectively. Meanwhile, the serum triglyceride level in the RDGM group was 26.3% lower than that in the RTGM group. The weight-loss effect in the RDGM group was accompanied by a significant decrease in the white adipose tissue (WAT) index. The RDG intervention did not significantly change the antioxidant and anti-inflammatory properties of the rapeseed oil in vivo. The RDG diet improved the liver lipid metabolism abnormalities induced by a high-fat diet, leading to decreased liver damage index values (AST and ALT). Additionally, compared to that in the RTGM group, the expression of the adipogenic genes *PPAR-γ* and *DGAT* decreased in both the liver and intestine by 21.7% and 16.7% and by 38.7% and 47.2%, respectively, in the RDGM group. Further, most lipolytic genes in BAT showed no significant change after the RDG intervention. This implies that RDG regulates lipid metabolism by altering the expression of adipogenic genes in the liver, intestine, and adipose tissue, thereby reducing the accumulation of WAT. Furthermore, the RDG diet enhanced gut flora diversity, increasing the relative levels of *unclassified Muribaculaceae* and decreasing the levels of *Dubosiella* and *Faecalibaculum* in the mouse gut, potentially accelerating lipid metabolism. Thus, a three-month RDG diet intervention in obese mice exhibited benefits in regulating the somatotype, serum obesity-related indices, gut flora structure, and lipid metabolism in the adipose tissue, liver, and intestine.

## 1. Introduction

Obesity (BMI > 30 kg/m^2^), as a phenotypic manifestation of abnormal or excessive fat accumulation, is characterized by enhanced adipogenesis and increased adipose tissue mass [1]. This sub-health state is caused by excessive energy intake and low energy expenditure, leading to pathological and physiological changes in humans. Obesity has become a serious health problem as it increases the risk of chronic diseases, such as cardiovascular disease, hypertension, type 2 diabetes, and certain types of cancer. Evidence shows that the prevalence of obesity is increasing globally, with an estimated 4 billion people expected to be obese by 2035 [2]. Considering the wide range of complications and the increased metabolic burden caused by obesity, preventing obesity is essential to regulating physical and mental health. The imbalance of energy intake over energy expenditure leads to lipid accumulation in adipose tissue [3]. White adipose tissue (WAT) plays a key role in storing triacylglycerol (TAG) in times of energy excess and secreting adipokines that are important in regulating glycolipid metabolism [4]. The excessive expansion of WAT directly causes obesity [5]. Brown adipose tissue (BAT) and beige adipose tissue, known as one type of adaptive thermogenic adipose tissue, dissipate energy in the form of heat and offer the therapeutic potential to counteract obesity and metabolic disorders [6]. BAT produces heat through non-oxidative uncoupling, which dissipates energy via non-shivering thermogenesis, increasing adipocyte glucose uptake and lipid metabolism [7]. In addition to its thermogenic functions, BAT can also regulate energy metabolism in mice [8]. Thus, decreasing the amount of WAT and increasing the amount of BAT is crucial to accelerating lipid metabolism and preventing obesity.

As one of the three most important nutrients for human beings, oils provide essential fatty acids and are important carriers enabling the absorption of nutrients such as vitamins in vivo. However, the current dietary structure of the modern population tends to include excessive consumption of edible oils, a significant contributing factor to obesity. The main component of edible oil, TAG, significantly contributes to obesity. After ingestion, TAG is easily resynthesized into TAG chylomicrons in the small intestine, which then accumulate in adipose tissue, increasing the metabolic burden and adversely affecting fat metabolism [9]. Diacylglycerol (DAG) is a minor component of natural oil formed by esterification between one molecule of glycerol and two molecules of fatty acids, and it has been recognized as a potential healthy substitute for traditional TAG-based oil. The main isomer of DAG, *sn*-1,3 DAG, is rarely resynthesized into TAG or TAG chylomicrons after digestion in the intestinal epithelium due to its unique structure. Instead, the digestive products of DAG are mostly utilized for energy and lipid metabolism regulation. DAG has been found to lower serum lipids [10], improve insulin sensitivity [11], reduce abdominal fat [12], prevent blood clots, and alleviate diseases related to lipid and glucose metabolism, such as cardiovascular disease [12]. DAG has also been found to reduce visceral fat in overweight people [13]. Additionally, DAG emulsion has been shown to promote the release of fatty acids in the intestine and have good digestive properties [14]. However, the molecular mechanism of DAG intake with regard to WAT and BAT accumulation and fat metabolism in vivo has rarely been studied.

Rapeseed oil, the leading domestic vegetable oil in China, is rich in a large amount of healthy unsaturated fatty acids and has unique properties making it suitable for the food industry [15]. However, as obesity and cardiovascular problems due to the excessive consumption of vegetable oils are now prevalent, research into healthier functional oils is essential. Rapeseed diacylglycerol oil (RDG) is among the functional oils with the greatest potential to replace traditional rapeseed oil in unhealthy modern dietary structures that involve a high lipid intake. The purpose of this study was to compare the ability of RDG and rapeseed triacylglycerol oil (RTG) to ameliorate syndromes associated with obesity in obese mice; thus, we designed an RDGM group (8-week HFD + 12-week RDG diet) and an RTGM group (8-week HFD + 12-week RTG diet). Another aim was to explore the anti-obesity effect of the long-term intake of RDG oil. Hence, we compared the body weight growth rate and lipid metabolic burden in an HFD group (20-week HFD) and an RDG group (20-week RDG diet). In this research, RDG was found to have anti-obesity effects in an obese mouse model, and the long-term RDG intervention prevented increases in body fat and lipid accumulation. This study investigated the effect of dietary RDG intake on lipid metabolism and adipocyte accumulation. The somatotype, serum markers of obesity-related indices, liver damage, adipose tissue weight, fat metabolism gene expression, and gut flora changes in the mice were compared after the RTG and RDG diet interventions. This study lays a foundation for further investigations into the mechanism by which DAG prevents obesity.

## 2. Material and Method

### 2.1. Feeding Preparation

RDG oil (50.88% RDG and 47.07%RTG) and RTG oil (94.99%RTG and 3.98%RDG) were obtained from Fastco Biotech Co., Ltd. (Hangzhou, China). RDG was prepared from rapeseed oil and glycerol catalyzed with Novozyme 435 (Candida antarctica lipase B) from Novozyme (China) Biotechnology Co., Ltd. (Guangzhou, China). After molecular distillation to remove monoglycerides and free fatty acids from the crude product, the crude product was then deodorized with stripping to obtain RDG oil. The proportion of *sn*-1,3 DAG and *sn*-1,2 DAG in RDG oil was 66:34. RTG oil was obtained from natural rapeseed oil. The liquid chromatograms of the acylglycerol composition and the gas chromatograms of the fatty acid composition of RDG and RTG oil are shown in Figure S1. And the analysis of acylglycerol composition and the fatty acid composition of RDG and RTG oil are presented in Table S1. The diet table of all groups is listed in Table 1. The oil accounts for 45% of the energy in the diet except for the Control group. The control and HFD feed includes 5.4% and 44.8% (energy) from both soybean oil and lard, respectively. The lipid of the RTG and RDG diets was instead of the same energy ratio of RTG oil and 50% RDG oil.

### 2.2. Animal Models and Study Design

Male C57BL/6J mice at 6 weeks of age were randomly divided into five groups (*n* = 8) named Control (control diet, 20 weeks), High-fat group (HFD diet, 20 weeks), RTGM group (8 weeks HFD diet + 12 weeks RTG diet), RDGM group (8 weeks HFD diet + 12 weeks RDG diet), and RDG (RDG diet, 20 weeks). Food intake was measured on a per-cage basis every day throughout this study. The organ index and the abdominal fat rate were calculated according to the following equations:

(1) $$organ\;index(\%)=\frac{organ\;weight}{body\;weight}\times 100\%$$

Mice were fed for 20 weeks and slaughtered overnight. The mice in this experiment were intraperitoneally injected with 50 mg/kg B.W. sodium pentobarbital before the sacrifice. Serum was collected from eyeballs and centrifuged. Then, the serum was frozen at −80 °C for analysis. Half of the liver, intestine, and colon tissues were also preserved at −80 °C for subsequent protein analysis. The remaining was stored in 10% formalin for hematoxylin–eosin staining after rats were sacrificed. Meanwhile, the fecal samples in the colons were collected, snap-frozen in liquid, and stored at −80 °C. The animal experiments in this study were approved by the Institutional Animal Care and Use Committee of Jinan University, and all the animal procedures were performed in accordance with the Guidelines for Care and Use Committee of Jinan University (Ethics application number: IACUC-20230217-01; Application date: 17 February 2023).

### 2.3. Determination of Serum Biochemical Indexes

When the obese mice model was completed (After 8 weeks of high-fat diet feeding), serum fasting glucose and ketone were measured per three weeks and six weeks using a glucometer made with a multifunctional blood glucose meter (VivaChek Laboratories, Inc., Hangzhou, China). The serum total cholesterol (TC), triglyceride (TG), high-density lipoprotein (HDL), low-density lipoprotein (LDL), malondialdehyde (MDA), and total superoxide dismutase (T-SOD) indexes were determined using a Hitachi 7600 automatic bioanalyzer (Hitachi, Tokyo, Japan). Aspartate aminotransferase (AST) and alanine aminotransferase (ALT) were then detected, and their assay kits were provided by Jiancheng Biological Engineering Co., Ltd. (Nanjing, China).

### 2.4. ELISA Assay for Inflammatory Cytokines Analysis

For the analysis of all cytokines, serum was prepared with centrifugation of whole blood at 1500× *g* for 10 min and stored in aliquots at −80 °C. The concentrations of tumor necrosis factor-α (TNF-α), interleukin-6 (IL-6), and interleukin-1β (IL-1β) in serum were assessed using an enzyme-linked immunosorbent assay (ELISA) according to the manufacturer’s instructions (Bio-Swamp Life Science, Shanghai, China). Then, samples were thawed and filtered for analysis using mouse-specific ELISAs following the manufacturer’s instructions. A Luminex 200 plate reader equipped with xPOnent software (Version 4.3, University of Rochester Flow Cytometry Core) was used to determine the median optical intensity for each analyte. The concentration of each analyte was calculated using the corresponding standard curve fit to a 5-parameter logistic equation. For all ELISAs, absorption was measured at 450 nm using a multi-well plate reader (AMR-100, Aosheng, Suzhou, China). Curve fitting and sample concentration calculations were conducted with Gen5 software (Version 2.07, Biotek, Winooski, VT, USA).

### 2.5. Hematoxylin and Eosin (H&E) Staining 

The liver, kidney, intestine, and adipose tissue in 10% formalin were embedded in paraffin, and 4 μm-thick sections were loaded on Superfrost™ Plus slides (Thermo Fisher Scientific, Waltham, MA USA). After rehydration of the samples with 100–70% ethanol solution, hematoxylin and eosin were used for staining different tissue samples, respectively. These slides were then photographed using a Nikon eclipse Ti-U microscope (Tokyo, Japan).

### 2.6. qRT-PCR

Total RNA from the intestine, liver, and BAT tissues was extracted using TRIzol reagent with a tissue homogenizer (Invitrogen Corporation Life Technologies, Carlsbad, CA, USA). The isolated RNA was processed using lithium chloride (Sangon Bio, Shanghai, China) and sodium acetate (Sangon Bio, Shanghai, China). The normalized RNA was calculated using a NanoDrop 2000C Spectrophotometer (Thermo Scientific, USA) and reverse-transcribed using a PrimeScript RT Reagent Kit with gDNA Eraser (TaKaRa Bio. Inc., Beijing, China). The primer sequences used for PCR are shown in Table 2. The relative expression levels of fat metabolism genes were measured using the 2^−ΔACT^ method.

### 2.7. 16S rDNA Sequencing of Gut Microbiota

The total genomic DNA of fecal bacteria was extracted using the TGuide S96 Magnetic Soil/Stool DNA Kit (TIANGEN, Beijing, China) according to the manufacturer's instructions. Twenty-five DNA samples (five mice were randomly selected from each experimental group) were selected for further analysis. The integrity of the DNA was assessed with agarose gel electrophoresis. The genomic DNA was used as a template for PCR amplification. Universal primers 515F and 806R were used for the PCR amplification of the V4 hypervariable regions of 16S rRNA genes (515F, 50-GTGYCAGCMGCCGCGGTAA-30; 806R, 50-GGACTACNVGGGTWTCTAAT-30). The generated DNA pool was then paired-end sequenced (2 × 250) on a Novaseq 6000 platform (Illumina, San Diego, CA, USA) at Biomarker Technologies Co., Ltd. (Beijing, China). 

### 2.8. Gut Flora Analysis

We analyzed the sequencing raw data using the Quantitative Insights into Microbial Ecology software package (version 2021.6). USEARCH (version 10.0) was employed to cluster sequences into operational taxonomic units (OTUs) with a similarity of over 97%. The taxonomy of each OTU representative sequence was analyzed using a Ribosomal Database Project Classifier v.2.2 trained on the database Greengene_2013_5_99 and Silva (Release132) with 0.6 confidence values as cutoff. All statistical analyses were performed using the R program (Version S-PLUS) and ade4 package (http://pbil.univ-lyon1.fr/ADE-4/, accessed on 16 November 2023) [16]. Alpha diversity calculation includes six metrics: OTUs, Ace, Chao1, Simpson, Shannon, and Coverage indices. The Shannon index was generated based on these metrics. Principal component analyses (PCA) with the two receiver mice groups at different time points as instrumental variables (interclass PCA) were computed and statistically assessed with a Monte Carlo rank test to observe their net effect on the scattering of the microbiota of different mice. Interclass PCA allows highlighting the combinations of variables that maximize variations observed between qualitative variables (e.g., specific responder-receiver (RR)/NRR groups). The Wilcoxon test was applied to assess the statistical significance of the bacterial composition between the different samples.

### 2.9. Statistical Analysis

The results were presented as mean and standard deviation (mean ± SD) using the SPSS 22.0 statistical software (IBM Corporation, Armonk, NY, USA) to perform one-way ANOVA between-group differences determined with Tukey analysis. When the *p*-value was less than 0.05, the independent test data of each group were considered statistically significant (*p* < 0.05).

## 3. Results

### 3.1. Apparent Change Characterization of the Mouse Somatotype after RDG Intervention

**Figure 1:**
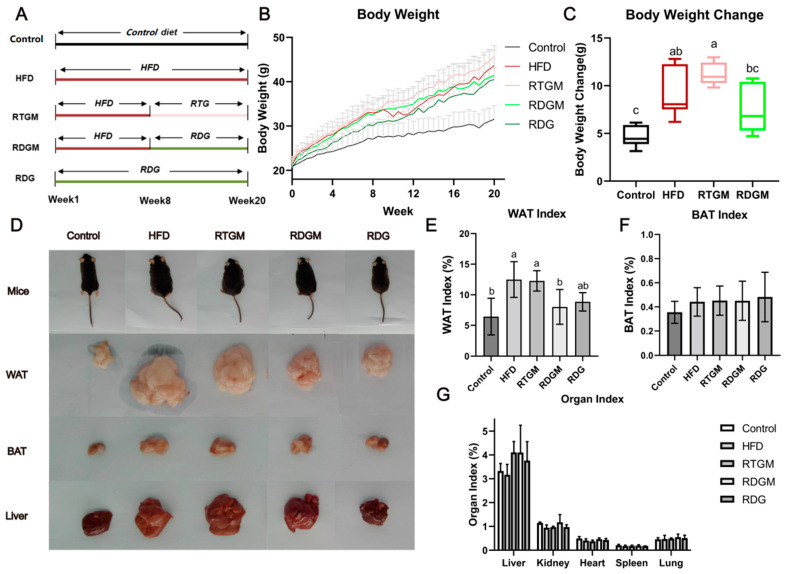
Group design (**A**), body weight during 20-week treatment period (**B**), body weight change between 8th and 20th week (**C**), body type and organs at 20th week (**D**), WAT index at 20th week (**E**), BAT index at 20th week (**F**)**,** and organ index at 20th week (**G**). Results are expressed as mean ± SD (*n* = 8). Means with the different letters are significantly different (*p* < 0.05). Note: HFD, high-fat diet; RDG, rapeseed diacylglycerol oil; RTG, rapeseed triacylglycerol oil; RDGM, rapeseed diacylglycerol oil intervention in obese model; RTGM, rapeseed triacylglycerol oil intervention in obese model; WAT, white adipose tissue; BAT, brown adipose tissue.

The group design for this study is presented in Figure 1A. For all groups other than the Control group, oil accounted for 45% of the energy in the mice’s diet. After 8 weeks of high-fat diet feeding, the average body weight of the mice had increased by approximately 20% compared to that in the Control group (Figure 1B), indicating that an obese mouse model had been successfully constructed. Then, the mice in the RTGM and RDGM groups were fed the RTG and RDG diets, respectively, while the diets in the other three groups remained the same. After 7 days of the intervention, the weight gain rate in the RDGM group was slower than that in the RTGM group, and the difference in body weight between these two groups gradually increased over time. From week 8 to the end of the trial, the average body weight gain in the RTGM group was 11.22 ± 1.18 g, while the average body weight gain in the RDGM group was 7.64 ± 2.63 g, indicating that a DAG diet can alleviate lipid accumulation in vivo (Figure 1C). The somatotypes of the RDGM and RDG mice were thinner (Figure 1D) than those of the HFD and RTGM mice. The morphology of the liver in the RTGM group was relatively bigger than that in the HFD and RDGM groups, while the RDG and Control groups showed similar liver morphologies. In addition, due to lipid accumulation in the livers of the HFD and RTGM groups, the liver index (liver weight/body weight) was higher in these two groups than in the other three groups. The WAT index (WAT weight/body weight) significantly dropped in the RDGM and RDG groups compared to the HFD and RTGM groups (Figure 1E), which is consistent with the WAT morphology across the different groups (Figure 1D). The WAT index values for the RTGM and RDGM groups were 12.27 ± 1.66% and 8.01 ± 2.84%, respectively. However, the phenotypic trends of the liver and BAT were not in line with the results for the liver index and BAT index, which both showed no significant difference between the RDGM and RTGM groups (Figure 1F,G). This was due to the increased body weight of mice in the RTGM group. In addition, there was no significant difference in the kidney, heart, lung, or spleen index values across all groups. The above results indicate that an RDG intervention in obese mice can inhibit weight gain, control body shape, and reduce liver lipid accumulation and the WAT index.

### 3.2. Fasting Serum Glucose and Ketones

**Figure 2:**
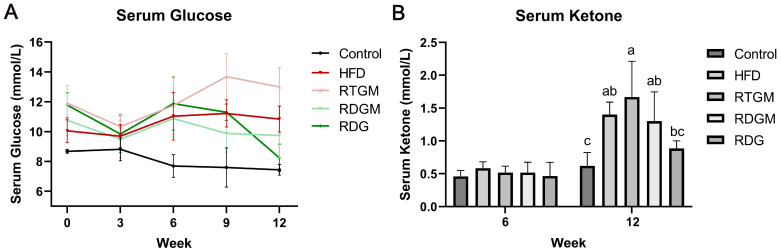
Fasting serum glucose (**A**) and fasting serum ketone (**B**) change in the 12 weeks after the establishment of the obese mice model. Results are expressed as mean ± SD (*n* = 8). Means with the different letters are significantly different (*p* < 0.05). Note: HFD, high-fat diet; RDG, rapeseed diacylglycerol oil; RTG, rapeseed triacylglycerol oil; RDGM, rapeseed diacylglycerol oil intervention in obese model; RTGM, rapeseed triacylglycerol oil intervention in obese model.

Obesity decreases insulin sensitivity, leading to a higher serum glucose level. Meanwhile, the oxidative metabolism of fat is enhanced in obese patients, resulting in a higher ketone content in their bodies. Prolonged elevated ketone levels can cause ketoacidosis in patients with type 2 diabetes. Measuring the serum ketone content of mice can thus reflect the oxidative decomposition of fat and the safety of RDG [17]. Figure 2A,B show the fasting serum glucose levels and fasting serum ketone levels of the mice at different time points. At the end of the experiment, the serum fasting glucose level was increased in the RTGM and HFD groups and decreased in the RDGM and RDG groups. The serum fasting glucose levels in the RTGM and RDGM groups were 13.0 ± 1.3 mmol/L and 9.7 ± 1.5 mmol/L, respectively, showing a significant difference in these two groups. In the 8th week of the diet intervention, the serum ketone levels in the RDGM and RDG groups were about 0.5 mM, with no significant difference between the groups. In the 20th week, there was no significant difference in serum ketones between the Control group and the RDG group, while the serum ketone levels in the RTGM and RDGM groups were 1.8 ± 0.4 mmol/L and 1.3 ± 0.5 mmol/L, respectively. The fasting serum ketone levels in the RDGM and RDG groups were lower than those in the RTGM and HFD groups. The above results indicate that a long-term high-fat diet increased the serum fasting glucose levels and serum fasting ketone levels in the mice. However, the RDG intervention alleviated these increases.

### 3.3. Serum Indices of Lipid Metabolism, Oxidative Stress, and Inflammatory Regulation

**Figure 3:**
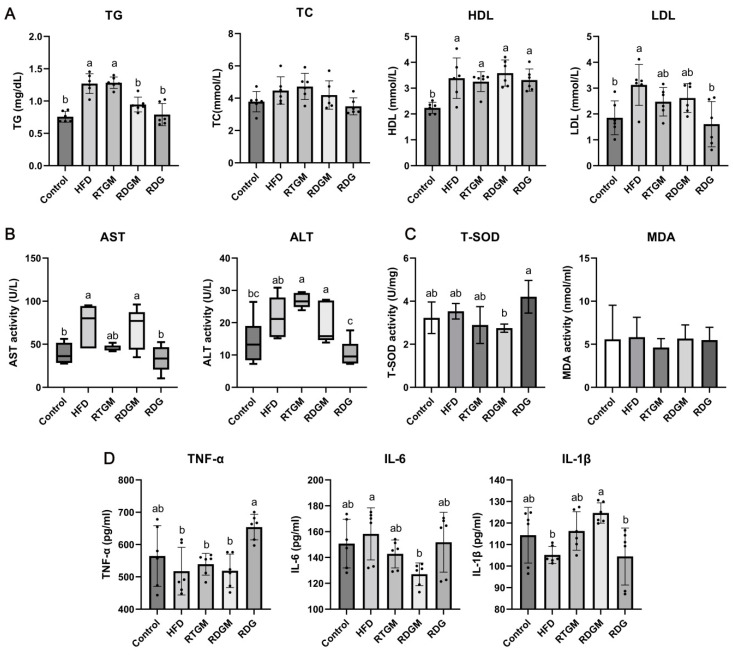
Serum biochemical indexes at 20th week. Lipid accumulation indexes measured with kits (**A**); liver injury degree indexes measured with kits (**B**); oxidative stress factor activity measured with kits (**C**)**,** and immune-inflammatory factor content measured with ELISA (**D**). Results are expressed as mean ± SD (*n* = 8). Means with different letters are significantly different (*p* < 0.05). Note: HFD, high-fat diet; RDG, rapeseed diacylglycerol oil; RTG, rapeseed triacylglycerol oil; RDGM, rapeseed diacylglycerol oil intervention in obese model; RTGM, rapeseed triacylglycerol oil intervention in obese model; TG, triacylglycerol; TC, total cholesterol; HDL, high-density lipoprotein; LDL, low-density lipoprotein; AST, aspartate aminotransferase; ALT, alanine aminotransferase; MDA, malondialdehyde; T-SOD, total superoxide dismutase; TNF-α, tumor necrosis factor-α; IL-6, interleukin-6; IL-1β, interleukin-1β.

The effects of RDG on lipid accumulation and cholesterol metabolism were then explored (Figure 3A). Compared with that in the HFD and RTGM groups, the TG content in serum was significantly decreased in the RDGM and RDG groups in the 20th week. The TG levels in the RTGM and RDGM groups were 1.3 ± 0.1 mg/dL and 0.9 ± 0.1 mg/dL, respectively. The TC content was slightly decreased in the RDGM and RDG groups (4.2 ± 0.9 mmol/L and 3.5 ± 0.5 mmol/L, respectively) compared to the RTGM group (4.7 ± 0.8 mmol/L), with no significant difference between the RDGM and RTGM groups. HDL and LDL are important lipoproteins that affect the cholesterol levels in serum. HDL is an anti-atherosclerotic lipoprotein that transports cholesterol from extrahepatic tissues to the liver for metabolism and excretion by bile. High plasma levels of HDL are inversely associated with the risk of cardiovascular disease. In contrast, LDL, which carries cholesterol from liver cells to tissue cells, is positively correlated with the risk of cardiovascular disease [18]. There was no significant difference in HDL content between the RTGM and RDGM groups, while the RDG group had a lower LDL content. Taken together, although there was no significant difference between the RDGM and RTGM groups in terms of HDL and LDL levels, the RDGM intervention slightly decreased the TC level. Additionally, the serum TG level in the RDGM group was 26.3 ± 3.3% less than that in the RTGM group, meaning that the RDG intervention significantly reduced the TG levels in serum.

The liver is the main organ for lipid synthesis, transportation, and metabolism. Excessive lipid accumulation in liver tissue causes abnormal levels of metabolic enzymes, leading to disordered lipid metabolism. Elevated AST and ALT levels in serum indicate hepatocyte damage and degeneration, serving as markers of liver damage [19]. A lower serum ALT content was observed in the RDGM group (19.0 ± 6.2 U/L) compared to the RTGM group (26.8 ± 2.2 U/L) according to the quartile content (Figure 3B), but there was no significant change in AST content across all the high-fat diet intervention groups. The lowest values of AST and ALT were observed for the RDG group. The oxidative metabolism of lipids can affect the oxidative stress microenvironment in vivo, and the excessive accumulation of lipids can lead to chronic inflammation. Therefore, the levels of oxidative stress and inflammatory factors in serum were also measured. There was no significant difference in the MDA level among any of the groups, and the RDG group had higher T-SOD activity than the other groups (Figure 3C). There was no significant difference between the RTGM and RDGM groups regarding the content of pro-inflammatory TNF-α. IL-6 and IL-1β are two proteins associated with inflammation, immune activation, and cell differentiation. The RDGM group had a significantly lower IL-6 content compared to the HFD group. Meanwhile, the RDGM group showed a significantly higher IL-1β content compared to the HFD group. However, there was no significant difference between the RDGM and RTGM groups. Thus, replacing RTG with RDG in the diet did not significantly affect the levels of multiple inflammatory and antioxidant indices overall, but it was beneficial in regulating lipid metabolism and negative effects on the liver (Figure 3D).

### 3.4. The Effect of RDG on Pathology and Adipogenic Genes of the Liver–Gut Axis

**Figure 4:**
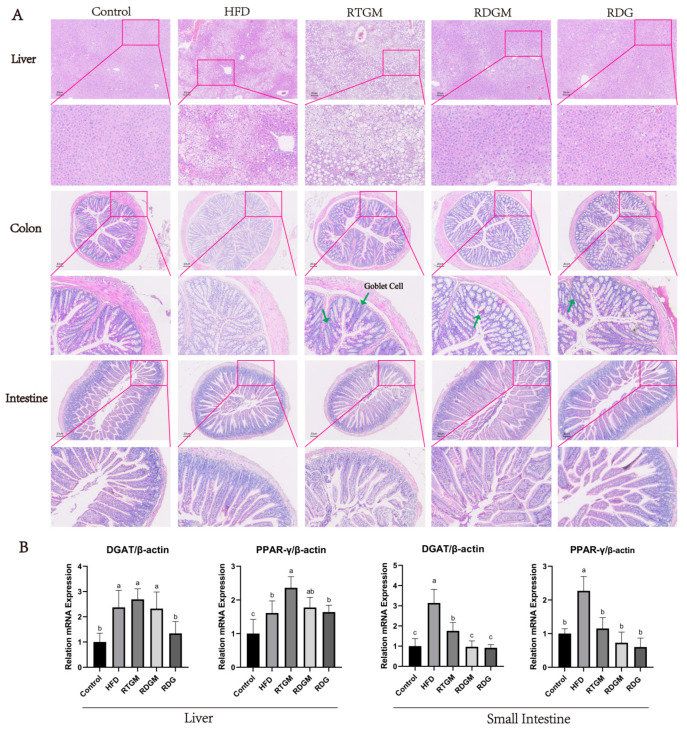
Hematoxylin–eosin staining (200×) of liver section, colon section, intestine tissue (**A**) and the expression levels of lipogenic genes (*DGAT* and *PPAR-γ*) in liver and intestine with real-time PCR at 20th week (**B**). Results are expressed as mean ± SD (*n* = 8). Means with different letters are significantly different (*p* < 0.05). Note: HFD, high-fat diet; RDG, rapeseed diacylglycerol oil; RTG, rapeseed triacylglycerol oil; RDGM, rapeseed diacylglycerol oil intervention in obese model; RTGM, rapeseed triacylglycerol oil intervention in obese model.

The cell morphology and lipid droplet size in the liver reflect the degree of metabolic burden in liver tissue, while the morphology of villi in the intestine and colon indicates the effect of RDG on the gastrointestinal tract. Figure 4A shows H&E-stained mouse liver, intestine, and colon tissues. In the liver sections, individual hepatocytes were complete with clear boundaries in the RDGM, RDG, and Control groups. The hepatocytes of these groups were intact with undamaged nuclei, exhibiting similar sizes and round shapes, and were evenly arranged around the central vein. In contrast, the HFD and RTGM groups displayed large cell gaps and numerous fat droplets aggregating into larger fat bubbles, indicating severe liver damage and fatty liver symptoms. This phenomenon was consistent with the high lipid content observed in the liver tissue (Figure 1D). The fat cells were vacuole-shaped and evenly arranged in the kidneys of all groups (Figure S3). There were fewer fat droplets and well-defined cellular spaces with no lesions or necrosis in the colon. Additionally, the content of goblet cells in the colon tissue was higher in the RDGM group than in the RTGM group. There were no significant differences in the intestine in any of the groups (Figure 4A). The RDG diet intervention resulted in beneficial lipid metabolism characteristics in the liver and intestinal sections.

Diacylglycerol acyltransferase (DGAT) catalyzes the final and rate-limiting step in the acyl-CoA-dependent biosynthesis of TAG, playing a crucial role in adipogenesis [20]. Peroxisome proliferator-activated receptor-γ (PPAR-γ) has also been proven to enhance fatty acid storage and TAG synthesis. The mRNA levels of the adipogenic genes *DGAT* and *PPAR-γ* were down-regulated in the RDGM group (relative levels: 2.3 ± 0.7 and 1.8 ± 0.3) in liver tissues compared with the RTGM group (relative levels: 2.7 ± 0.4 and 2.3 ± 0.4) (Figure 4B). Meanwhile, the RDGM intervention significantly down-regulated the expression of *DGAT* (relative level: 1.0 ± 0.3) in the intestine, as compared with the RTGM intervention (relative level: 1.8 ± 0.4). The relative levels of *PPAR-γ* in the RDGM and RTGM groups were 0.7 ± 0.4 and 1.2 ± 0.2, respectively, in intestinal tissues. Overall, the RDG intervention down-regulated the transcription of adipogenic genes in both the intestine and liver, with a more pronounced effect in the intestine.

### 3.5. The Effect of RDG on Lipid Metabolism in Adipose Tissue

**Figure 5:**
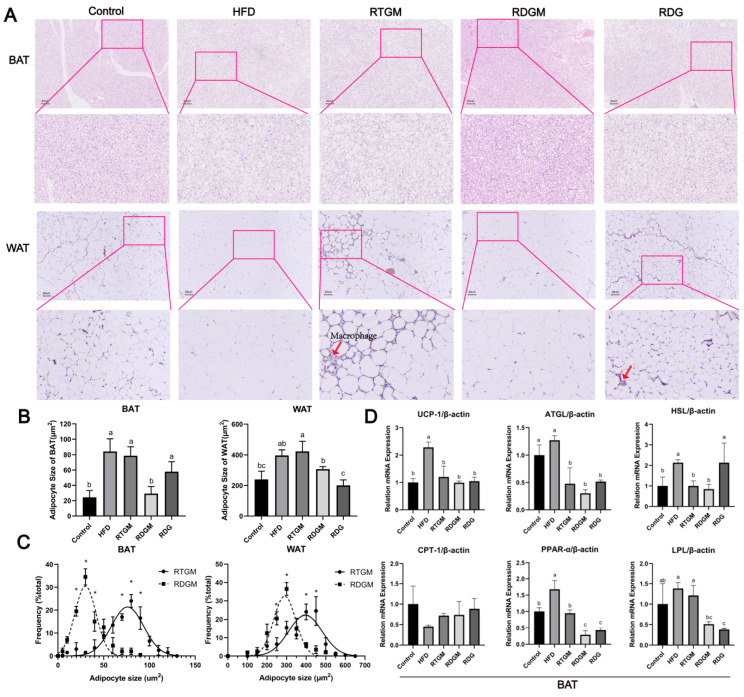
Hematoxylin–eosin staining (200×) (**A**); adipocyte size (**B**); adipocyte size frequency in RTGM and RDGM groups of WAT and BAT (**C**), and expression of adipolysis genes (*UCP-1*, *ATGL*, *HSL*, *CPT-1*, *PPAR-α*, and *LPL*) in BAT with real-time PCR at 20th week (**D**). Results are expressed as mean ± SD (*n* = 8). Means with different letters are significantly different (*p* < 0.05) and * *p* < 0.05. Note: HFD, high-fat diet; RDG, rapeseed diacylglycerol oil; RTG, rapeseed triacylglycerol oil; RDGM, rapeseed diacylglycerol oil intervention in obese model; RTGM, rapeseed triacylglycerol oil intervention in obese model; WAT, white adipose tissue; BAT, brown adipose tissue.

WAT comprises mature adipocytes and the stromal vascular fraction, which includes pre-adipocytes, fibroblasts, endothelial cells, stem cells, and immune cells. BAT is mainly characterized by multilocular lipid droplets and abundant mitochondria, enabling it to produce heat through non-shivering thermogenesis [21]. H&E staining of fat sections was performed to observe the effect of RDG on adipocyte hypertrophy and evaluate the contribution of RDG to lipid accumulation [22]. The adipocyte sizes in both the WAT and BAT of the RDGM group (307.3 ± 16.5 μm^2^ and 29.2 ± 9.2 μm^2^, respectively) were significantly smaller than those in the RTGM group (423.2 ± 65.0 μm^2^ and 78.8 ± 11.3 μm^2^, respectively) (Figure 5A,B), indicating that the RDG diet intervention significantly reduced adipocyte hypertrophy. Regarding macrophages, some were observed in the RTGM group, fewer were observed in the RDG group, and none were observed in the RDGM group. A quantitative analysis of the adipocyte size in adipose tissue was then performed (Figure 5C). The frequency distribution curve of the RDGM group was steeper than that of the RTGM group, indicating a more concentrated adipocyte size distribution in the RDGM group. Therefore, the RDG intervention had benefits in reducing and concentrating the adipocyte size in both WAT and BAT.

Lipid metabolism relies not only on lipid synthesis but also on lipolysis to prevent lipid accumulation. The lipolysis process in humans is predominantly carried out with BAT. Therefore, the effects of the RDG diet on the transcription of key genes involved in lipid metabolism in BAT were then explored (Figure 5D). Six lipolytic genes related to lipolysis and thermogenesis processes—*hormone-sensitivelipase* (*HSL*), *uncoupling protein 1* (*UCP-1*), *adipose triglyceride lipase8 *(*ATGL*), *carnitine palmitoyltransferase I* (*CPT-1*), *peroxisome proliferator-activated receptor-α* (*PPAR-α*), and *lipoprotein lipase* (*LPL*)—were selected to evaluate the effect of RDG. Among these genes, the relative levels of *PPAR-α* and *LPL* were significantly down-regulated in the RDGM group (0.3 ± 0.1 and 0.5 ± 0.1, respectively) compared with the RTGM group (0.9 ± 0.1 and 1.2 ± 0.2, respectively). No obvious difference was observed in the transcription of *UCP-1*, *ATGL*, *HSL*, or *CPT-1* between the RTGM and RDGM groups. Thus, the RDGM intervention significantly down-regulated the expression of some lipolytic genes in obese mice. Meanwhile, *HSL* was highly expressed in the long-term RDG intervention (RDG group, 2.1 ± 0.9).

### 3.6. Spearman Analysis and Changes in Gut Flora Due to the RDG Intervention 

**Figure 6:**
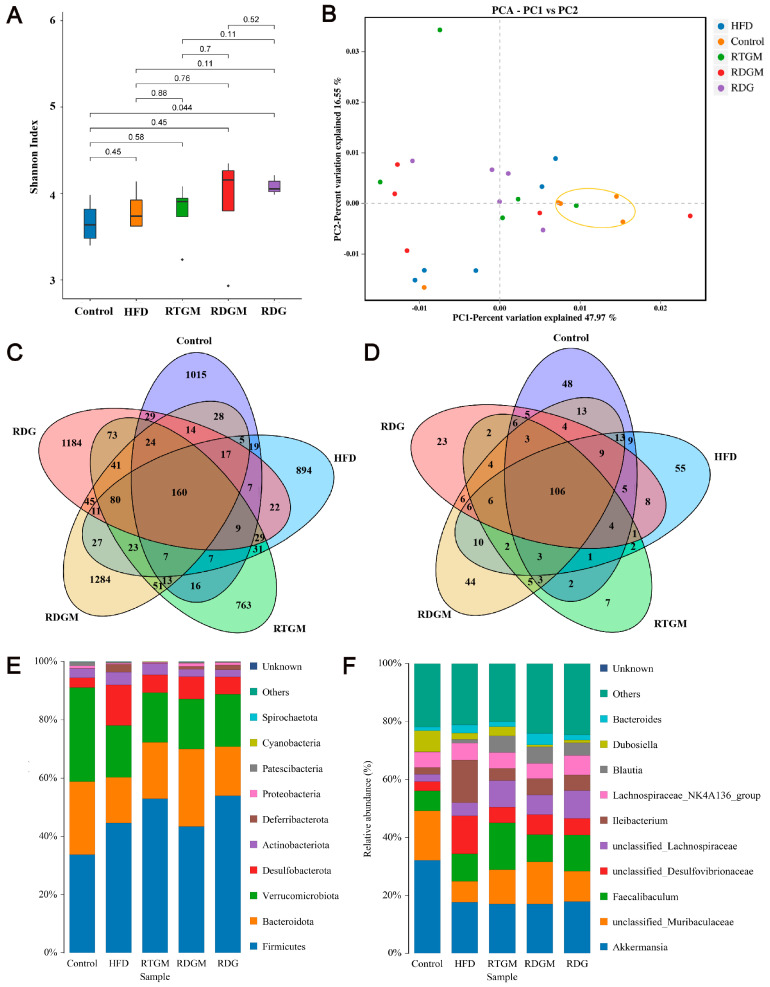
Cecal microflora structure of rats in each group. Shannon Index of species distribution at the genus level (**A**); β-diversity assessed with PCA analysis based on weighted UniFrac distances (**B**); Venn diagram of cecal microflora at the OUT level (**C**); Venn diagram of cecal microflora at the genus level (**D**); histogram of species distribution at the phylum level (**E**)**,** and histogram of species distribution at the genus level (**F**). Results are expressed as mean ± SD (*n* = 5). Note: HFD, high-fat diet; RDG, rapeseed diacylglycerol oil; RTG, rapeseed triacylglycerol oil; RDGM, rapeseed diacylglycerol oil intervention in obese model; RTGM, rapeseed triacylglycerol oil intervention in obese model.

The diversity of the gut microbial community is associated with metabolic health factors such as lipid metabolism. The α diversity can represent the abundance of species within each microbial community in individual gut flora. The α diversity index of the gut flora was observed at the genus classification level (Figure 6A). The Shannon index values for the RDGM, RTGM, and RDG groups were 3.9 ± 0.6, 3.8 ± 0.3, and 4.1 ± 0.1, respectively. The β diversity indicates the degree of difference in the gut microbial communities of different individuals. After the high-fat diet intervention, all groups showed similar microbial diversity and relative abundance, resulting in no significant clustering of sample spacing between the groups. The percentage variations for PC-1 and PC-2 were 46.97% and 16.55%, respectively, indicating no significant difference in β diversity among the high-fat diet groups. There were 160 OTUs shared by the cecal bacteria across all five groups. Additionally, the RDGM and RDG groups had 1284 and 1184 unique OTUs, respectively, that were not found in the other groups (Figure 6C). Similarly, the RTGM and RDGM groups had different cecal bacteria at the phylum level (Figure 6D). A low gut Firmicutes/Bacteroidetes (F/B) ratio is generally considered a biomarker of obesity [23]. It was found that the F/B value of the RDGM group was lower than that of the RTGM group (Figure 6E). The RDG group also had a relatively high F/B value, and its relative *Dubosiella* level was quite low, just like in the RDGM group. At the genus level, the dominant flora in all groups included *Akkermansia*, *unclassified Muribaculaceae*, and *Faecalibaculum*. Compared with the RTGM group, the RDGM group had a more obvious increase in *unclassified Muribaculaceae* and a decrease in *Dubosiella* and *Faecalibaculum* (Figure 6F). The results showed that the α species diversity increased, and the flora structure changed after the RDG intervention. Simultaneously, dominant species and genera were identified after the RDG intervention. This aided in identifying the flora characteristics related to obesity and provided a clearer understanding of how RDG alters the gut flora structure.

**Figure 7:**
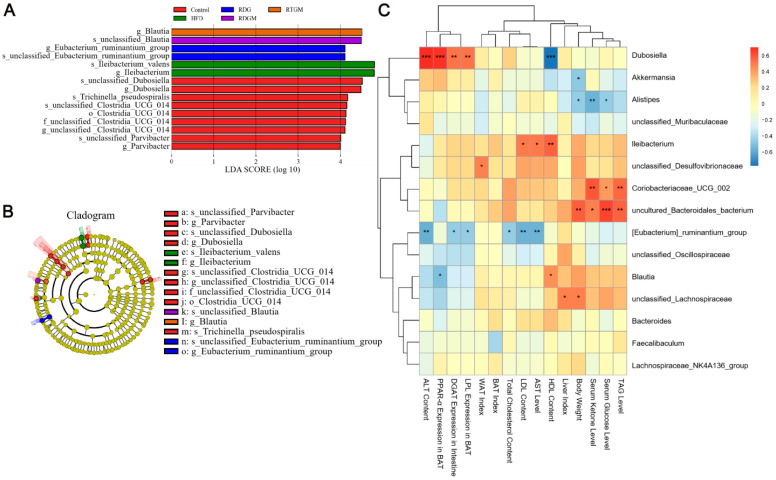
LEfSe analysis and Spearman analysis between gut flora and obesity-related index of rats, (**A**) LEfSe biomarker graph, (**B**) LEfSe_biomarkers_cladogram, and (**C**) Heatmap of gut flora in metabolism with Spearman analysis in genus level (*n* = 5). * *p* < 0.05; ** *p* < 0.01; *** *p* < 0.001. Note: HFD, high-fat diet; RDG, rapeseed diacylglycerol oil; RTG, rapeseed triacylglycerol oil; RDGM, rapeseed diacylglycerol oil intervention in obese model; RTGM, rapeseed triacylglycerol oil intervention in obese model; TAG, triacylglycerol; WAT, white adipose tissue; BAT, brown adipose tissue; AST, aspartate aminotransferase; ALT, alanine aminotransferase; HDL, high-density lipoprotein; LDL, low-density lipoprotein.

LEfSe was used to analyze comparisons among multiple groups to find species with significant differences in abundance. To further analyze the regular changes in the gut flora after the RDG intervention and the relationships between dominant flora and obesity-related indices, LEfSe biomarker and Spearman analyses were then performed (Figure 7). In Figure 7A, *Blautia* in the RTGM group was more abundant at the genus level, while *Blautia* in the RDGM group was more abundant at the species level. This may be due to the fact that the fatty acid composition of the rapeseed oil did not differ in the RTGM and RDGM groups; therefore, the beneficial flora was similar. Additionally, the RTGM group exhibited higher abundance than the RDGM group in *Clostridia_UCG_014*, *Dubosiella*, *Parvibacter*, and *Trichinella_pseudospiralis*. The rapeseed oil diet reduced *lleibacterium*, with higher abundance in the HFD group, and *Eubacterium_ruminantium*, with higher abundance in the RDG group (Figure 7B). We then performed a Spearman analysis between the major flora and multiple obesity-related markers. *Dubosiella* was found to have a strong positive correlation with adipolysis gene expression, lipogenic gene expression, and lipid damage level indices. *Uncultured Bacteroidales bacterium*, *Coriobacteriaceae UCG 002*, and *Dubosiella* were found to have a strong positive correlation with serum anti-obesity-related indices like serum TAG, serum glucose, and serum ketone levels. *[Eubacterium]_ruminantium_group* had a strong negative correlation with liver damage levels (Figure 7C). After the RDG intervention, the abundance of *Blautia* in the RDGM and RTGM groups was dominant on different flora levels, and the RDGM group displayed a decreased abundance of multiple flora. In addition, the correlation analysis between different flora and different indices was helpful to analyze the effect of these flora against obesity factors.

## 4. Discussion

After the establishment of the high-fat model, the RDG treatment resulted in a lower body weight increase rate, serum TG level, serum TC level, serum fasting glucose level, and serum fasting ketone level, as compared with the RTG treatment. The somatotype and serum analysis results proved that the RDG intervention has potential benefits in preventing obesity and regulating lipid metabolism [12]. However, the daily intake in the HFD group decreased briefly after the establishment of the high-fat model, which may be due to the HFD diet being very greasy, resulting in increasing satiety and, eventually, anorexia (Figure S2). The hydrophilicity endowed by the unique structure of RDG can reduce the greasy feeling of the diet [24]. Studies have shown that DAG intake over 3 months can significantly lower the serum glucose level [12], and this decline in serum glucose levels has been observed both with and without HFD interventions. The RDG intervention groups showed a lower increase in serum ketone content, indicating that the RDG diet imposed less of a metabolic burden than the other diets. This is mainly due to the greater need in the RTGM group to decompose adipocyte tissue as a source of energy after fasting; thus, the fasting serum glucose level in the RTGM group was higher than that in other groups. A previous study found that a diet with 90% DAG (by weight) from rapeseed oil was able to reduce the total fat mass in obese patients when compared with TAG intake [25], and alpha-linolenic-acid-enriched DAG (80% by weight) intake was found to be negatively correlated with the visceral fat area in obese individuals [26]. This study further found that the mechanism by which DAG reduces body fat may be via a reduction in the WAT index. In addition, when the mice were slaughtered after fasting, the levels of lipolysis in the WAT in the RDG group were observed to have decreased (Figure 5), which also corresponded to lower fasting serum ketone levels in the RDG and RDGM groups than in the RTGM group [27,28]. In this research, the RDG group had a relatively low serum LDL content, which may relate to the expression of apolipoprotein B (ApoE/B). DAG has been proven to reduce the mRNA expression of ApoE/B, the main component of LDL, and greatly affect the content of LDL in serum [29]. Therefore, this benefit may not apply to improvements in the cholesterol lipoprotein content with a short-term DAG intervention. Inflammatory factors relate not only to an increased risk of metabolic disorders such as dyslipidemia and insulin resistance but also to thermogenic responses by suppressing brown/beige adipogenesis [30]. *sn*-1,2 DAG was shown to reduce serum TNF-α and IL-6 levels [31]. In this study, the RDG and RDGM groups showed slightly higher response values of inflammatory factors than the RTGM group. This is mainly due to the oxidation stability of DAG being less than that of RTG [32] and *sn*-1,2 DAG accounting for a small proportion of natural DAG. Therefore, the conversion of unsaturated fatty acids to saturated fatty acids due to the oxidation instability of DAG may lead to a slight increase in inflammatory factors. However, there was no significant difference among the groups, indicating that RDG did not interfere with the inflammatory microenvironment in vivo.

DAG has been shown to reduce the content of visceral fat in animal experiments [29] and decrease the area of visceral fat in human experiments [33]. The weight loss that has been observed in obese subjects after DAG intake may be associated with a decrease in the visceral fat area [26]. In this study, the WAT index was significantly reduced in the RDG treatment groups, indicating that RDG is beneficial in reducing lipid storage. Obese individuals without metabolic complications have smaller adipocytes in WAT, accompanied by lower degrees of inflammation and fibrosis, compared with their unhealthy counterparts. This is because hypertrophy triggers the development of severe metabolic diseases [21]. Improving adipose tissue heat production is effective in improving obesity-induced insulin resistance [34]. DAG has been found to significantly regulate mitochondrial metabolic thermogenesis [35]. PPAR-α plays an important role in enzyme β-oxidation and promotes the synthesis of LPL, catalyzing the lipolysis of TAG in lipoproteins into free fatty acids. Meanwhile, LPL catalyzes the decomposition of TAG in the core of chylomicrons into fatty acids for tissue oxidation, energy supply, and storage. RDG down-regulated the expression of these two thermogenic genes, indicating that RDG prevented the lipolysis process in BAT. HSL encodes fatty acid hydrolase, which promotes the decomposition and release of lipids. The higher expression level of *HSL* in the RDG group may be attributed to DAG regulating lipid metabolism through the regulation of the Lipe and Fabp4 genes [36]. UCP-1 is involved in non-shivering adaptive thermogenesis in BAT and generates heat by regulating proton transport in mitochondria, decoupling ATP synthesis and oxidized phosphoric acid [37]. ATGL is the initial enzyme in the process of TAG esterolysis. There was no significant difference in *ATGL* transcription among the RTGM, RDGM, and RDG groups. However, there was a significant trend of the adipogenesis process in the liver and small intestine being inhibited after RDG intervention (Figure 5). The expression levels of *DGAT* and *PPAR-γ* in the intestine and liver were decreased in the RDGM group compared with the RTGM group, suggesting that the RDG intervention inhibited the adipogenesis process in this high-fat diet model. Dietary DAG stimulates β-oxidation and lipid-metabolism-related gene expression, with intestinal lipid metabolism making the predominant contribution to the effects of DAG [35]. However, in this study, the lesser lipid accumulation in adipocyte tissue after the RDG intervention resulted in a lower lipolytic degree compared to that in the RTGM group. The fasting serum ketone index also corroborated the hypothesis that the ketone level was decreased in the long-term-intake RDG group. Therefore, RDG tended to limit the adipogenesis process.

Gut flora affect the host's energy acquisition and storage from their diet. The species diversity of intestinal flora in obese people is significantly lower than that in non-obese people [23]. The relative abundance of lipid-metabolism-related genera in the RDG intervention groups increased significantly, indicating that the proportion of lipid metabolism by the gut flora was increased. At the phylum level, the F/B value is related to obesity. Studies have shown that the F/B value of obese mice is higher than that of thinner mice, and the same relationship was also found in obese people [38]. However, some human experiments showed the opposite trend [39]. Rapeseed oil has a certain function of accelerating lipid metabolism by increasing the relative proportion of *Blautia* [40]. In this study, *Blautia,* the dominant flora in both the RDGM and RTGM groups, was found to reduce visceral fat, indicating that RDG did not hinder RTG’s beneficial effects regulating the gut flora [41]. This is mainly due to the RDG and RTG diets sharing a similar fatty acid composition, further verifying the anti-obesity effect of rapeseed oil. In addition, *Blautia* has complex effects on lipid metabolism; it is positively correlated with obesity-promoting indicators such as DGAT and ALT but also positively correlated with obesity-preventing indicators such as LPL and PPAR-α. *Desulfobacterota*, which can reduce sulfate to hydrogen sulfide and has an undesirable effect on intestinal inflammation, was greatly reduced in all rapeseed oil intervention groups [42]. This phenomenon may stem from the functional properties of the fatty acids and other active components present in rapeseed oil [15]. Among the gut flora at the genus level, the species *Faecalibaculum rodentium* might contribute to the fasting fat mass accumulation associated with gut-specific glut1 inactivation [23]. *Faecalibaculum* was shown to be significantly less abundant in the RDGM group than in the RTGM group. A reduction in *Faecalibaculum* is associated with the prevention of obesity [23]. Probiotic-mediated anti-obesity effects are also linked to members of the *Bacteroides* genus [23], and a higher proportion of *Bacteroides* was found in the RDGM group compared with the RTGM group. This indicated that RDG can alleviate the adverse changes in the intestinal microbiota induced by an HFD. *Coriobacteriaceae_UCG_002* is positively correlated with obesity and the serum TG content [43], indicating that this bacterium can promote obesity. The proportion of *Dubosiella* in the gut flora showed a significant reduction in both the RDGM and RDG groups, which is conducive to preventing microbial malregulation and promoting the expansion of short-chain-fatty-acid-producing bacteria [44]. *Dubosiella* was also positively correlated with lipogenic gene expression and liver damage indices, suggesting that a reduction in *Dubosiella* is beneficial to lipid metabolism. *[Eubacterium]_ruminantium_group* members act as beneficial short-chain-fatty-acid-producing bacteria [45] and showed a significant negative correlation with serum liver injury indicators and LDL content. Given that RDG has the potential to decrease liver damage and regulate cholesterol metabolism, this relationship suggests that RDG intake can regulate lipid metabolism by releasing more short-chain fatty acids.

## 5. Conclusions

The evidence presented herein shows that the weight-loss effect of the RDG intervention was accompanied by a significant decrease in the WAT index, fasting serum TG level, and fasting serum glucose level. Additionally, RDG improved the liver lipid metabolism abnormalities induced by the high-fat diet, decreased the adipocyte size, and resulted in a more concentrated adipocyte size distribution in WAT and BAT. At the same time, the expression of adipogenic genes was decreased in the liver and intestine. Therefore, RDG regulates lipid metabolism by altering the expression of lipid synthesis genes in the liver, intestine, and adipose tissue and reducing the accumulation of WAT. In addition, RDG enhanced the diversity, increased the relative levels of unclassified *Muribaculaceae*, and decreased the levels of *Dubosiella* and *Faecalibaculum* in the mice’s gut flora; these changes are related to lipid metabolism. In conclusion, an RDG-enriched high-fat diet has benefits in regulating the somatotype, serum obesity-related indices, gut flora structure, and lipid metabolism in the adipose tissue, liver, and intestine.

## Figures and Tables

**Table 1:** The diet table of all groups.

Raw Materials (g/kg)	Control Diet	HFD Diet	RTG Diet	RDG Diet
Oil	Soybean oil + Lard 40	Soybean oil + Lard 202.5	RTG oil 202.5	RDG oil 202.5
Casein	200	200	200	200
L-Cysteine	3	3	3	3
Corn starch	315	72.8	72.8	72.8
Maltodextrin	35	100	100	100
Sucrose	350	167.8	167.8	167.8
Cellulose	50	50	50	50
Mixed mineral	10	50	50	50
Mixed vitamins	10	10	10	10

Note: The energy value of all diets is 4.73 kcal/g; RTG, rapeseed triacylglycerol oil; RDG, rapeseed diacylglycerol oil; TAG, triacylglycerol; DAG, diacylglycerol; MAG, monoacylglycerol; FFA, free fatty acid. Mixed minerals include sucrose, potassium citrate tripotassium, monohydrate, dicalcium phosphate, calcium carbonate, sodium chloride, magnesium sulfate, heptahydrate, and other minor minerals. Mixed vitamins include vitamin A, vitamin B, vitamin D, vitamin E and vitamin K; the control diet was bought from Beijing Huafukang Biotechnology Co., Ltd. (Beijing, China), and other diets were bought from the Guangdong Medical Laboratory Animal Center.

**Table 2:** Primer sequences used in this study.

Gene	Forword (5′ to 3′)	Reverse (5′ to 3′)
PPAR-γ	TGTGGACCTCTCCGTGATGG	GGTTCTACTTTGATCGCACTTTGG
PPAR-α	GGATGTCACACAATGCAATTCGCT	TCACAGAACGGCTTCCTCAGGTT
DGAT	AGTGGCAATGCTATCATCATCGT	AAGGAATAAGTGGGAACCAGATCA
CPT-1	GCACTGCAGCTCGCACATTACAA	CTCAGACAGTACCTCCTTCAGGAAA
UCP-1	AAGACAGAAGAGCATAGCATTCAC	CCAGTCATACACTCCCACCTC
HSL	CTGCTGACCATCAACCGAC	CGATGGAGAGAGTCTGCA
LPL	GTACCTGAAGACTCGCTCTC	AGGGTGAAGGGAATGTTCTC
ATGL	AGTGAGTGGCTGGTGAAAGGT	CGCCTTGCTGAGAATCACCAT

## Data Availability

The original contributions presented in the study are included in the article, further inquiries are available upon reasonable request from the corresponding author.

## Supplementary Materials

The following supporting information can be downloaded at: https://www.mdpi.com/article/10.3390/nu16132003/s1, Figure S1: The liquid chromatograms of acylglycerol composition and gas chromatograms of fatty acid composition of RDG oil (a,b) and RTG oil (c,d); Figure S2: The change of average diet intake; Figure S3: The change of average diet intake; Table S1: The acylglycerol composition and fatty acid composition of RTG oil and RDG oil.
