# Supplementary material for: The Lipid-Metabolism-Associated Anti-Obesity Properties of Rapeseed Diacylglycerol Oil
Source: Nutrients. 2024 Jun 24;16(13):2003. doi: 10.3390/nu16132003 (PMC11243274; doi:10.3390/nu16132003)
Supplement: Supplementary file 1 [file nutrients-16-02003-s001.zip › nutrients-2991683-supplementary.pdf]

Table S1. The acylglycerol composition and fatty acid composition of RTG oil and RDG oil.

Constituents (%)	FFA	MAG	DAG	TAG	C16:0	C18:1	C18:2	C18:3	Others
RTG	1.03	ND	3.98	94.99	4.08	62.44	19.15	9.03	5.10
RDG	0.70	1.35	50.88	47.07	3.71	59.6	22.24	8.15	4.67

RTG, rapeseed triacylglycerol oil; RDG, rapeseed diacylglycerol oil; TAG, triacylglycerol; DAG, diacylglycerol; MAG, monoacylglycerol; FFA, free fatty acid.

(**a**)

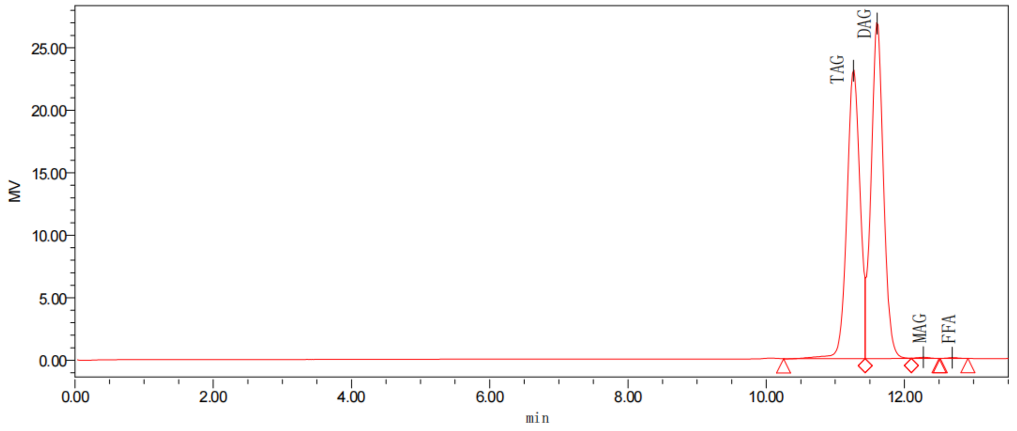

(**b**)

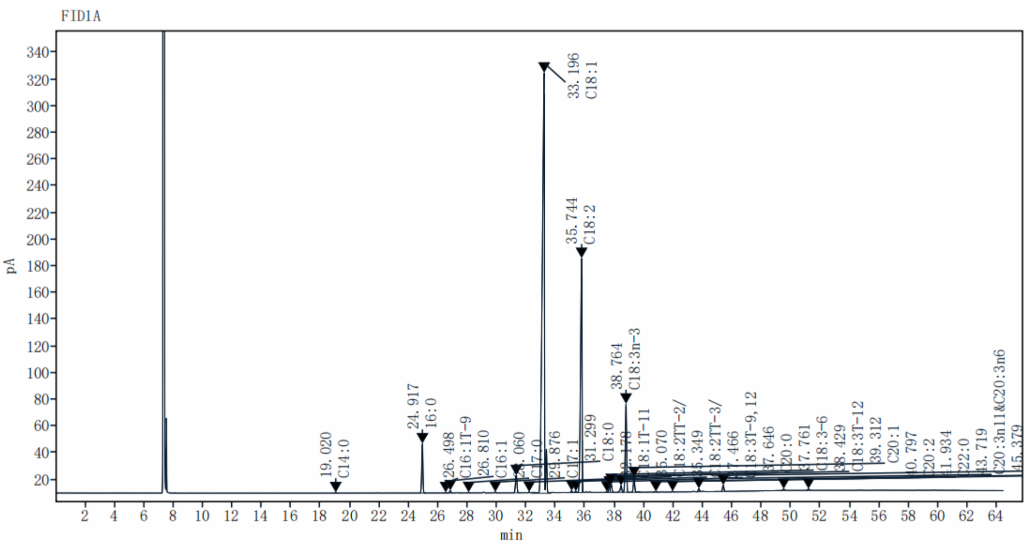

(**c**)

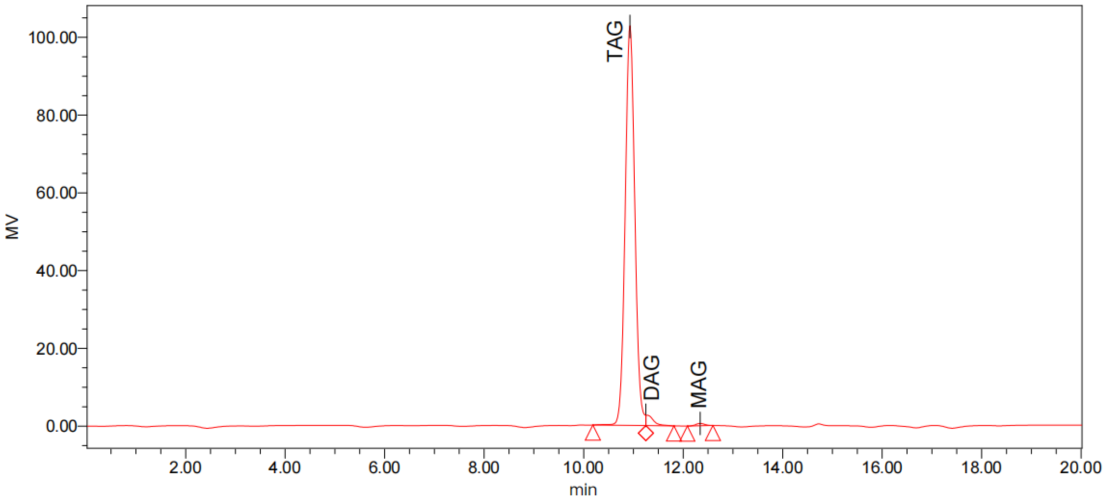

(**d**)

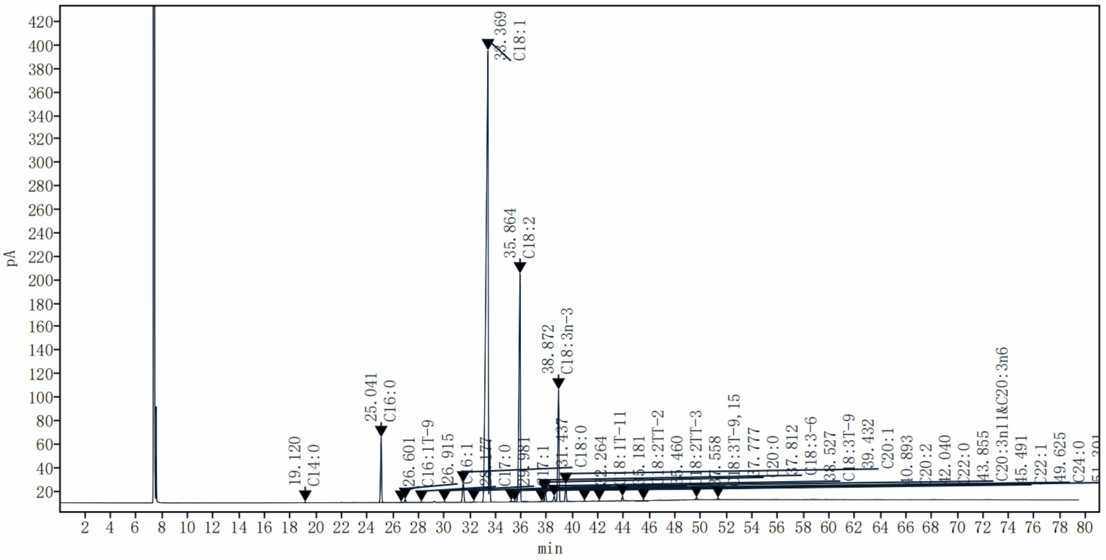

Figure S1. The liquid chromatograms of acylglycerol composition and gas chromatograms of fatty acid composition of RDG oil (**a**,**b**) and RTG oil (**c**,**d**).

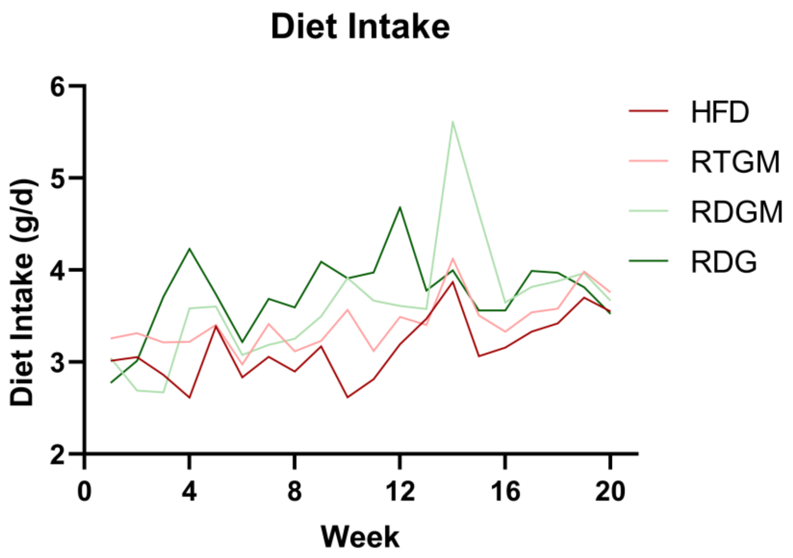

Figure S2. The change of average diet intake.

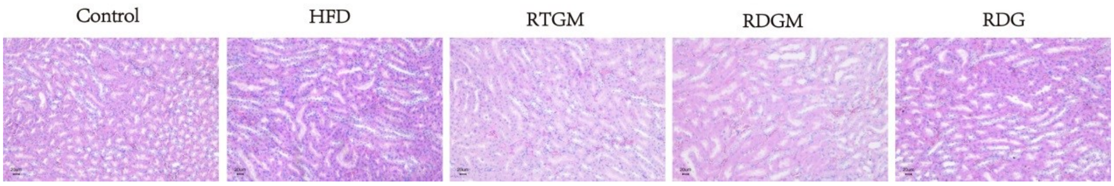

Figure S3. The Hematoxylin–eosin staining of histological section (200×) of kidney.
